# Differences in Beliefs About Cholesterol-Lowering Medications Among the Visegrad Group Countries: A Cross-Sectional Study

**DOI:** 10.3389/fpubh.2021.645043

**Published:** 2021-04-30

**Authors:** Klára Boruzs, Zita Fekete, Viktor Dombrádi, Gábor Bányai, Attila Nagy, Robert Horne, Klára Bíró

**Affiliations:** ^1^Department of Health Systems Management and Quality Management for Health Care, Faculty of Public Health, University of Debrecen, Debrecen, Hungary; ^2^Department of Behavioural Sciences, Faculty of Medicine, University of Debrecen, Debrecen, Hungary; ^3^Faculty of Public Health, University of Debrecen, Debrecen, Hungary; ^4^School of Pharmacy, University College London, London, United Kingdom

**Keywords:** beliefs about medicines questionnaire, necessity, concern, cholesterol lowering medication, Visegrad countries

## Abstract

**Background:** New cholesterol guidelines highlight more personalized risk assessments and new cholesterol-lowering drugs for people at the highest risk for cardiovascular disease. Adherence due to fear of and lack of trust in medications prevents treatment to provide better health outcomes.

**Objectives:** The aim of our study was to investigate the possible differences in the beliefs about the necessity and concerns regarding lipid-lowering drugs among the Visegrad Group countries.

**Methods:** The Beliefs About Medicines Questionnaire (BMQ-Specific) was used in our research. The responses of 205 Hungarian, 200 Slovak, 235 Czech, and 200 Polish participants, all taking cholesterol-lowering medications, were compared to each other.

**Results:** Hungarian participants' belief in the necessity of cholesterol-lowering drugs was significantly lower compared to the Slovak (*P* = 0.001), Czech (*P* = 0.037), and Polish (*P* < 0.001) participants. While no difference was observed between the Czech and Slovak responses (*P* = 0.154), both the Czech (*P* < 0.001) and Slovak (*P* = 0.006) respondents' belief regarding necessity was lower than that of the Polish. Regarding concerns, the only significant difference was observed between the Czech and the Polish respondents (*P* = 0.011).

**Conclusions:** While the beliefs about benefits (necessity) are most prominent among the Polish participants, except in comparison to Czech responses, the Visegrad Group countries do not differ considerably regarding their beliefs about the fear (concerns) of the treatment.

## Introduction

Cardiovascular mortality is more exalted in Central Eastern European countries (1) compared to the high-income member states in the European Union. In addition, the health status of the population of Central Eastern European countries is more unequal compared to other developed countries (2).

Socioeconomic differences play an important role regarding health inequalities (3). People with lower social status, wealth, and education often die earlier than those who are better off and better educated (4).

According to a recently published study, lower level of education was the most significant predictor of mortality in the Visegrad Group countries, namely, Poland, the Czech Republic, Slovakia, and Hungary. The lowest mortality rates by all causes of death were found in the regions of the Czech Republic, and the highest in Hungary. Despite the similar historical origin, various socioeconomic factors such as employment, poverty, and education seem to be different not only within the Visegrad Group countries, but within various regions of each country as well. Therefore, where citizens live within these countries can significantly influence their health (2).

Raised cholesterol is a risk factor for cerebrovascular and cardiovascular diseases. Every third ischemic heart disease and 2.6 million deaths per year can be attributed to high cholesterol worldwide. Overall, raised cholesterol is estimated to cause 29.7 million disability adjusted life years (1). The prevalence of raised cholesterol increases with the economic development of a region. According to the findings of the World Health Organization (WHO) in 2008, the European region had the highest elevated total cholesterol values compared to the other WHO regions, whereas the report suggests that the prevalence of raised cholesterol level in countries that joined the European Union after 2004 is lower than in the EU15 countries. Poland is the only exception where the prevalence of elevated cholesterol is as high as in the countries of Western Europe (5). Nevertheless, according to a 2018 Eurostat report, Eastern European countries, especially the countries of Visegrad Group, had the lowest cardiovascular morbidity. Within the group, Poland had the lowest (610.5/100,000 inhabitants), while Hungary had the highest (782.2/100,000) rates in 2015 (6).

The presence of organized public health has a long history in the Visegrad Group countries, and there is university-level public health education in all four countries. In addition to adequate public health services and the presence of competent doctors, it would be important to understand the behavior of the population and factors contributing to the way of behavior. One of these factors may be the individual's beliefs of health- or illness-related behavior. Beliefs are mental convictions that involve representational content and assumed veracity. Furthermore, beliefs are unquestioned representations of the world containing confidence in objects that the individual considers as veridical. Thus, beliefs influence attitudes, decisions, and behavior (7).

Horne and Weinman suggest (8) that chronically ill patients have general beliefs regarding medicine and specific beliefs about various kinds of prescribed medication. The specific beliefs can significantly influence adherence and decisions of patients (9–11). They also propose the effects of a cost–benefit assessment of specific beliefs and personal views of the patients. As a result, patients make judgements about the necessity (benefit) of their medication and concerns (cost) about its adverse effects (8). The relative balance of beliefs about necessity and concerns determines the behavioral intentions of the patients, insofar as the predominance of necessity (benefit) increases the likelihood of adherence behavior (8). According to the Health Belief Model by Rosenstock (12), the individual's evaluation of the possible outcomes is affected by perceived benefits and costs, and this evaluation influences future behavior.

Cholesterol treatment adherence is decisive to prevent cardiovascular diseases (13). Therefore, it is also worth taking into account further contributing factors like effects of social environment. The Theory of Planned Behavior by Ajzen (14) suggests that behavioral intentions can be predicted through the person's attitudes toward the behavior, subjective norms, and perceived control. Normative beliefs include social normative beliefs defined by the social environment. Potential reference groups are relevant, such as family members or friends but even the society at large has a significant influence on normative beliefs and, through this, on behavioral intentions (15). This is supported by a literature summary of Weber et al., which emphasizes the role of culture in decision making (16). Hence, normative beliefs influenced by cultural norms may contribute to intentions for adherence.

Taking these notions into account, and that there is a growing interest to investigate the various similarities and differences between the Visegrad Group countries (17–19), the aim of this study was to investigate the possible differences in the beliefs about necessity and concern regarding anticholesterol treatment between the Polish, Czech, Slovak, and Hungarian citizens taking cholesterol-lowering medications. Identifying similarities would mean that similar policies and best practices can be utilized to improve adherence in all four countries.

## Materials and Methods

### Study Design and Sample Size

Ethical approval was obtained from ethics committees in countries where non-interventional studies required it prior to data collection. The Scientific Research and Ethics Committee of the Medical Research Council (ETT TUKEB 55704-5/2017/EKU) in Hungary and the Ethics Committee of the Czech University Hospital Hradec Kralove (Ref. number: 201802 S15P) in the Czech Republic gave these approvals. The data collection was performed by the SZLEM Service L.P. in Hungary and by the Ceský Národní Panel Ltd. in Slovakia, the Czech Republic, and Poland. These companies have extensive experience in collecting data for market research purposes. The same data collection methods were applied in the four studied countries. The questionnaire used in our study was sent online to citizens from the companies' database. Those in the database had to give consent to be contacted for participating in various surveys. For each country, 1,000 adult citizens were asked to complete the questionnaire while taking into account representativeness by age, gender, and population of both the region and the settlement at a country level. As not everyone in the selected population was taking cholesterol-lowering drugs, a smaller sample was randomly selected, of which all participants were taking the chosen drug. Therefore, 205 Hungarian, 200 Slovak, 235 Czech, and 200 Polish responses were analyzed in this study.

### Measures

The Beliefs about the Medicines Questionnaire (BMQ) was used in our study (8). This questionnaire was originally written in English and consists of two sub-questionnaires, BMQ-Specific and BMQ-General. Because of our research aim, we only used the BMQ-Specific sub-questionnaire. This sub-questionnaire was created by Horne and colleagues to assess beliefs about medications that affect the treatment. It is made up of two scales, Specific-Necessity with five questions and Specific-Concerns with six questions. For each question, five possible answers could be given from “Strongly disagree” to “Strongly agree” (from 1 = strongly disagree to 5 = strongly agree). Depending on the distribution of data, the scores of both scales are determined by the mean or median value of the items. The subscale of Specific-Necessity investigates, on one hand, the patients' belief of the necessity of prescribed medication and, on the other hand, the subjective importance of regular medicine intake (20–22). Thus, a higher Specific-Necessity score means that the respondents have a stronger belief for the need to take the medication. The Specific-Concerns score means concerns about the negative effects of taking that specific medication (8).

The BMQ-Specific for cholesterol-lowering drugs was translated into all the four target languages of which all of them were deemed valid and reliable (23).

### Data Analysis

As a preliminary analysis, patients were categorized into groups based on their beliefs about medication (24, 25); these groups were created by splitting BMQ-Necessity and Concerns scores at the median value. Four categories were created with this method: “Skeptical,” “Ambivalent,” “Indifferent,” and “Accepting.” Respondents of the category “Indifferent” are neither convinced of its need nor concerned about, while “Ambivalent” of cholesterol-lowering medication means that the respondents are approving the necessity of cholesterol-lowering drugs but are also concerned about its possible adverse effects. “Accepting” cholesterol-lowering drugs means that responders are approving the necessity of cholesterol-lowering drugs while holding low concerns about its potential adverse effects. Respondents approving low necessity and high concern to cholesterol-lowering drugs were categorized into the “Skeptical” group. They hold doubts about personal need and increased concern about taking cholesterol-lowering drugs.

Descriptive statistics were performed to analyze the demographic characteristics of the respondents and the overall necessity and concerns scores for each country. These analyses included mean, standard deviation (SD), median, interquartile range (IQR), variance, skewness, and kurtosis. Chi-squared test and Kruskal–Wallis test were used to investigate if a significant difference exists between the countries regarding the various demographic data and the two scales. Univariate robust regression was applied to explore how gender, age, education level, marital status, perceived financial status, being a healthcare worker, perceived health status, having a chronic disease, and country of origin might influence the necessity and concerns scores. Finally, multivariate robust regression models were built in which all the demographic data were included as potential confounding factors. In each analysis, a *P*-value <0.05 was deemed significant. All the analyses were performed using Stata Version 13.0.

## Results

### Sample Characteristics

In Hungary (61.0%), Slovakia (55.5%), and the Czech Republic (59.1%), most participants were female, while in Poland, most of the participants were male (53.0%) (Table 1). In all countries, about more than half of the respondents claimed to have a high school education as their highest level of education (50.7–63.0%), and most of them were between the ages of 55–65 (38.0–61.7%). Also, in all four countries, the majority of participants were married and claimed to have a fair financial status. In addition, only a few of the participants were health workers. Most respondents stated having good health, but also having at least one chronic disease. There was a significant difference regarding gender, age, education, marital status, and perceived financial status between the countries (*P* < 0.001), but not for healthcare workers (*P* = 0.360), perceived health status (*P* = 0.467), and the number of chronic disease (*P* = 0.081).

**Table 1:** Demographic data of the respondents in the Visegrad Group countries.

	**Hungary**		**Slovakia**		**Czech republic**		**Poland**		***P*-value**
	***n***	**%**	***n***	**%**	***n***	**%**	***n***	**%**	
**Gender**									
Female	125	61.0%	111	55.5%	139	59.1%	94	47.0%	<0.001
Male	80	39.0%	89	44.5%	96	40.9%	106	53.0%	
**Age**									
18–24	0	0.0%	2	1.0%	1	0.4%	2	1.0%	<0.001
25–34	1	0.5%	6	3.0%	8	3.4%	11	5.5%	
35–44	10	4.9%	28	14.0%	15	6.4%	42	21.0%	
45–54	29	14.1%	58	29.0%	55	23.4%	55	27.5%	
55–65	121	59.0%	81	40.5%	145	61.7%	76	38.0%	
+65	44	21.5%	25	12.5%	11	4.7%	14	7.0%	
**Education**									
Primary school	30	14.6%	24	12.0%	63	26.8%	6	3.0%	<0.001
High school	104	50.7%	102	51.0%	132	56.2%	126	63.0%	
College	71	34.6%	74	37.0%	40	17.0%	68	34.0%	
**Marital status**									
Single	13	6.3%	25	12.5%	17	7.2%	17	8.5%	<0.001
Married or in a relationship	137	66.8%	146	73.0%	156	66.4%	147	73.5%	
Widow	19	9.3%	6	3.0%	8	3.4%	15	7.5%	
Divorced	35	17.1%	23	11.5%	54	23.0%	16	8.0%	
Lives separately	1	0.5%	0	0.0%	0	0.0%	5	2.5%	
**Financial status**									
Very good	1	0.5%	10	5.0%	6	2.6%	7	3.5%	<0.001
Good	21	10.2%	36	18.0%	63	26.8%	71	35.5%	
Fair	133	64.9%	110	55.0%	125	53.2%	81	40.5%	
Bad	34	16.6%	29	14.5%	32	13.6%	33	16.5%	
Very bad	13	6.3%	13	6.5%	8	3.4%	7	3.5%	
I don't know	3	1.5%	2	1.0%	1	0.4%	1	0.5%	
**Healthcare worker**									
Yes	14	6.8%	8	4.0%	11	4.7%	15	7.5%	0.360
No	191	93.2%	192	96.0%	224	95.3%	185	92.5%	
**Health status**									
Excellent	0	0.0%	0	0.0%	1	0.4%	1	0.5%	0.467
Very good	5	2.4%	10	5.0%	12	5.1%	8	4.0%	
Good	110	53.7%	119	59.5%	111	47.2%	108	54.0%	
Fair	70	34.1%	57	28.5%	84	35.7%	62	31.0%	
Poor	20	9.8%	14	7.0%	27	11.5%	21	10.5%	
**Chronic disease**									
Yes, but only one	78	38.0%	86	43.0%	84	35.7%	88	44.0%	0.081
Yes, more than one	120	58.5%	112	56.0%	144	61.3%	101	50.5%	
No	7	3.4%	2	1.0%	7	3.0%	11	5.5%	

### Descriptive Analysis of the Responses

Because of the non-normal distribution of the data, we used median scores to compare the answers between the four countries (Table 2). The Hungarian respondents had the lowest necessity score with 2.60, while the Polish participants had the highest score with 3.00. The respondents from the Czech Republic had the lowest score in concerns with 2.50, while the other three countries had the same scores with 2.67. According to the Kruskal–Wallis test, there was a significant difference regarding necessity between the countries (*P* < 0.001), but not for concerns (*P* = 0.235).

**Table 2:** Results regarding necessity and concerns of the Visegrad Group countries.

	**Necessity**				**Concerns**			
	**Hungary**	**Slovakia**	**Czech republic**	**Poland**	**Hungary**	**Slovakia**	**Czech republic**	**Poland**
Mean	2.56	2.85	2.81	3.08	2.71	2.67	2.59	2.75
SD	0.94	0.91	0.84	1.03	0.92	0.90	0.89	0.87
Variance	0.88	0.82	0.70	1.05	0.84	0.81	0.78	0.76
Skewness	0.43	0.05	0.05	−0.03	0.22	0.19	0.37	0.14
Kurtosis	2.92	2.60	2.84	2.48	2.69	2.49	2.61	2.53
Q1	1.80	2.20	2.40	2.50	2.17	2.00	1.83	2.08
Median	2.60	2.80	2.80	3.00	2.67	2.67	2.50	2.67
Q3	3.00	3.50	3.40	3.80	3.33	3.33	3.17	3.33
IQR	1.20	1.30	1.00	1.30	1.17	1.33	1.33	1.25
*P*-value	<0.001				0.235			

*SD, Standard deviation; Q, Quartile; IQR, Interquartile Range*.

**Figure 1:**
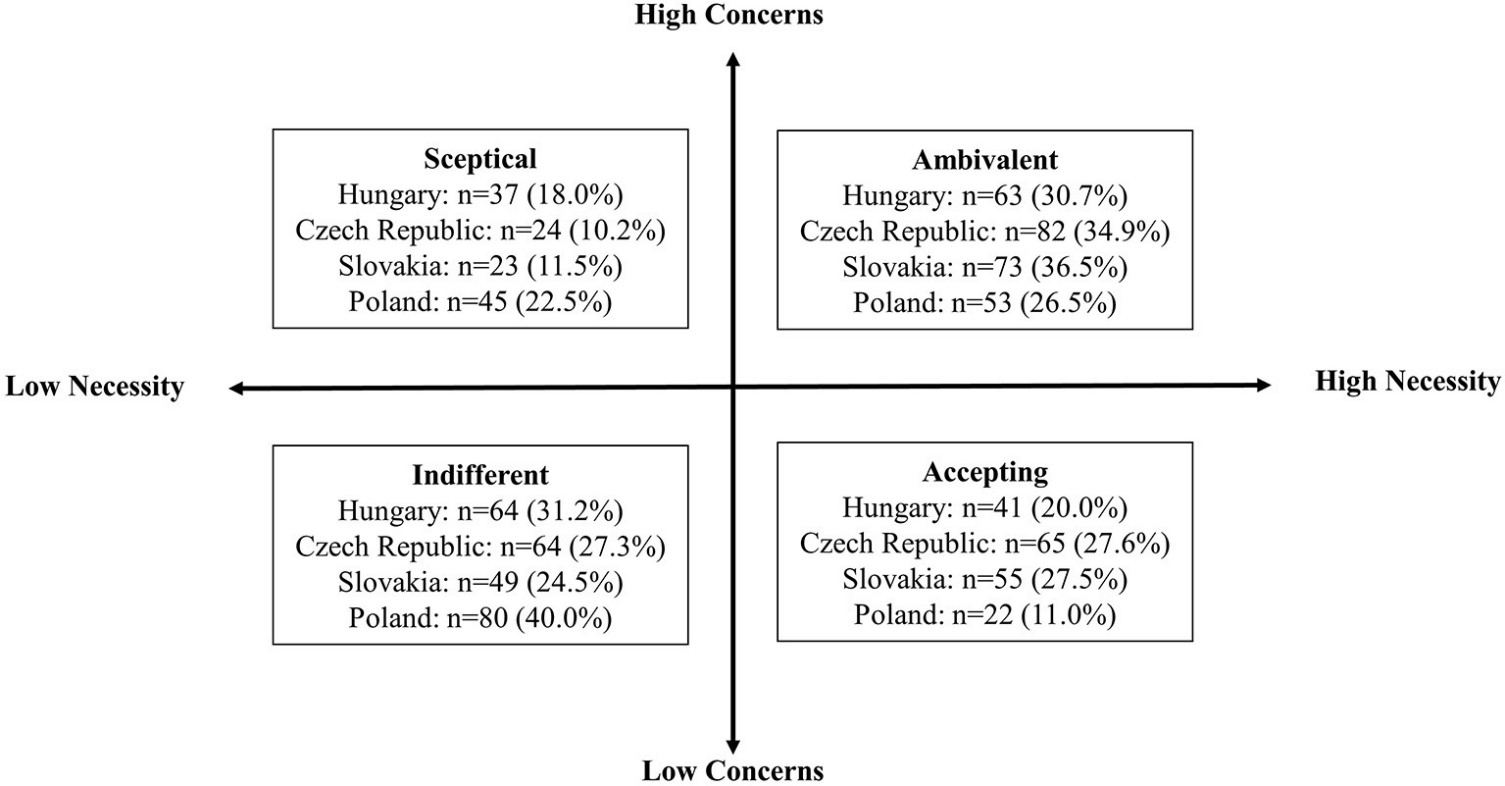
Proportion of the respondents allocated to each attitudinal group regarding cholesterol lowering drugs for each of the four countries.

Figure 1 shows the proportion of the sample allocated to each attitudinal group. Most Hungarian respondents were within the “Indifferent” (31.2%) and “Ambivalent” (30.7%) groups. The Czech and Slovak respondents were grouped similarly as a third of the sample were classified as “Ambivalent” (34.9 and 36.5%) regarding cholesterol-lowering medication, approving the necessity of cholesterol-lowering drugs but concerned about its potential adverse effects. The next group of respondents was “Accepting” (27.6 and 27.5%) of cholesterol-lowering drugs, approving the necessity of cholesterol-lowering drugs while holding low concerns about its potential adverse effects. A similar proportion was “Indifferent” (27.3 and 24.5%), neither convinced of its need nor concerned about taking it. The lowest proportion of respondents were found to be “Skeptical” (10.2 and 11.5%) to cholesterol-lowering drugs holding doubts about personal need and increased concern about cholesterol-lowering drugs. Most Polish respondents were within the “Indifferent” (40.0%) and “Ambivalent” (26.5%) groups.

### Comparative Analysis of the Responses

The results of the univariate and multivariate robust regression analysis regarding necessity are presented in Table 3. When considering demographic variables as confounding effects, participants who claimed to be in a good or better health status considered less necessary to take cholesterol-lowering treatment compared to those claiming a fair or poor health status (*P* = 0.006). For participants with higher or secondary education, qualifications also found it less necessary to take cholesterol-lowering medication to those who had a primary education (*P* < 0.001 in both cases). When comparing the four countries with each other, Hungarian participants significantly believed less in the necessity of cholesterol-lowering drugs compared to the Slovak (*P* = 0.001), Czech (*P* = 0.037), and Polish (*P* < 0.001) participants. While no difference was observed between the Czech and Slovak responses (*P* = 0.154), both the Czech (*P* < 0.001) and Slovak (*P* = 0.006) respondents' belief regarding necessity was lower than that of the Polish. This means that compared to the other three countries, the Polish participants believed most in the necessity of the cholesterol-lowering drug.

**Table 3:** Multivariate analysis of necessity within the Visegrad Group countries.

**Factors**		**Univariate analysis**				**Multivariate analysis**			
		**Coef**.	***P***	**95% CI**		**Coef**.	***P***	**95% CI**	
**I. Demographic data**									
Gender	Female/**Male**	0.04	0.528	−0.09	0.18	0.11	0.105	−0.02	0.24
Age	18–24/**35*****–*****44**	−0.20	0.661	−1.09	0.69	−0.22	0.619	−1.08	0.64
	25–34/**35*****–*****44**	−0.25	0.249	−0.68	0.18	−0.19	0.372	−0.61	0.23
	45–54/**35*****–*****44**	−0.08	0.495	−0.33	0.16	−0.05	0.700	−0.28	0.19
	55–65/**35*****–*****44**	−0.21	0.061	−0.43	0.01	−0.11	0.308	−0.33	0.10
	>65/**35*****–*****44**	−0.49	**0.007**	−0.85	−0.14	−0.34	0.054	−0.69	0.01
Education	Secondary/**Primary**	−0.31	**0.002**	−0.51	−0.12	−0.37	** <0.001**	−0.57	−0.17
	Higher/**Primary**	−0.39	** <0.001**	−0.60	−0.17	−0.41	** <0.001**	−0.63	−0.18
	Higher/***Second*****ary**	−0.07	0.344	−0.22	0.08	−0.03	0.669	−0.18	0.12
Marital status	Married or in a relationship/**Other**	0.13	0.092	−0.02	0.27	0.12	0.116	−0.03	0.26
Financial status	Fair/**Bad or very bad**	−0.07	0.421	−0.25	0.10	0.11	0.238	−0.07	0.29
	Good or better/**Bad or very bad**	−0.15	0.131	−0.35	0.05	−0.02	0.823	−0.24	0.19
	Good or better/**Fair**	−0.08	0.318	−0.24	0.08	−0.13	0.108	−0.30	0.03
Healthcare worker	Yes/**No**	0.04	0.770	−0.25	0.33	−0.02	0.885	−0.30	0.26
Health status	Good or better/**Fair or poor**	−0.30	** <0.001**	−0.43	−0.17	−0.22	**0.006**	−0.37	−0.06
Chronic disease	Has only one/***Non*****e**	0.16	**0.023**	0.02	0.30	0.08	0.279	−0.07	0.23
	Has multiple/***Non*****e**	−0.26	0.178	−0.65	0.12	−0.19	0.321	−0.57	0.19
	Has multiple/**Has only one**	0.42	**0.029**	−0.81	−0.04	−0.27	0.163	−0.66	0.11
**II. Countries**									
Hungary	Slovakia/**Hungary**	0.32	**0.001**	0.13	0.51	0.34	**0.001**	0.15	0.53
	Czech Republic/**Hungary**	0.29	**0.002**	0.11	0.47	0.20	**0.037**	0.01	0.39
	Poland/**Hungary**	0.57	** <0.001**	0.38	0.76	0.61	** <0.001**	0.41	0.80
Slovakia	Hungary/**Slovakia**	−0.32	**0.001**	−0.51	−0.13	−0.34	**0.001**	−0.53	−0.15
	Czech Republic/**Slovakia**	−0.03	0.745	−0.21	0.15	−0.14	0.154	−0.32	0.05
	Poland/**Slovakia**	0.25	**0.011**	0.06	0.44	0.27	**0.006**	0.08	0.46
Czech Republic	Hungary/**Czech Republic**	−0.29	**0.002**	−0.47	−0.11	−0.20	**0.037**	−0.39	−0.01
	Slovakia/**Czech Republic**	0.03	0.745	−0.15	0.21	0.14	0.154	−0.05	0.32
	Poland/**Czech Republic**	0.28	**0.003**	0.09	0.46	0.41	** <0.001**	0.22	0.60
Poland	Hungary/**Poland**	−0.57	** <0.001**	−0.76	−0.38	−0.61	** <0.001**	−0.80	−0.41
	Slovakia/**Poland**	−0.25	**0.011**	−0.44	−0.06	−0.27	**0.006**	−0.46	−0.08
	Czech Republic/**Poland**	−0.28	**0.003**	−0.46	−0.09	−0.41	** <0.001**	−0.60	−0.22

*Coef, Coefficient; CI, Confidence interval; P, significance of statistical test; P < 0.05 significance; Reference groups are underlined. The multivariate analysis includes all demographic variables as confounding effects. The values in bold are significant*.

The same results regarding concerns are reported in Table 4. Women were significantly more concerned about cholesterol-lowering medication than men (*P* = 0.002). Those who claimed to have at least good health status had lower concerns to those who claimed to have a fair or poor health status (*P* = 0.004). Respondents working in healthcare were more concerned about cholesterol-lowering treatment than those who did not (*P* = 0.013). Having multiple chronic disease also significantly increased concerns compared to those having none (*P* = 0.049), but not to those having only one (*P* = 0.110). After conducting the multivariate analysis between the four countries, the only significant difference was observed between the Czech and the Polish respondents (*P* = 0.011), in which the Polish were more concerned.

**Table 4:** Multivariate analysis of concerns within the Visegrad Group countries.

**Factors**		**Univariate analysis**				**Multivariate analysis**			
		**Coef**.	***P***	**95% CI**		**Coef**.	***P***	**95% CI**	
**I. Demographic data**									
Gender	Female/**Male**	0.18	**0.005**	0.06	0.31	0.20	**0.002**	0.07	0.33
Age	18–24/**35*****–*****44**	−0.19	0.663	−1.04	0.66	−0.12	0.777	−0.97	0.72
	25–34/**35*****–*****44**	−0.06	0.780	−0.47	0.35	−0.04	0.853	−0.45	0.37
	45–54/**35*****–*****44**	0.11	0.369	−0.13	0.34	0.11	0.328	−0.11	0.34
	55–65/**35*****–*****44**	0.05	0.624	−0.16	0.26	0.02	0.828	−0.19	0.24
	>65/**35*****–*****44**	−0.13	0.469	−0.47	0.22	−0.13	0.443	−0.48	0.21
Education	Secondary/**Primary**	−0.10	0.278	−0.29	0.08	−0.07	0.477	−0.26	0.12
	Higher/**Primary**	−0.08	0.413	−0.29	0.12	0.01	0.933	−0.21	0.23
	Higher/***Second*****ary**	0.02	0.797	−0.12	0.16	0.08	0.289	−0.07	0.23
Marital status	Married or in a relationship/**Other**	−0.01	0.861	−0.15	0.13	0.03	0.642	−0.11	0.17
Financial status	Fair/**Bad or very bad**	−0.25	**0.003**	−0.42	−0.08	−0.14	0.127	−0.31	0.04
	Good or better/**Bad or very bad**	−0.36	** <0.001**	−0.55	−0.17	−0.21	0.056	−0.42	0.01
	Good or better/**Fair**	−0.10	0.183	−0.26	0.05	−0.07	0.392	−0.23	0.09
Healthcare worker	Yes/**No**	0.35	**0.011**	0.08	0.62	0.35	**0.013**	0.08	0.63
Health status	Good or better/**Fair or poor**	−0.26	** <0.001**	−0.39	−0.14	−0.22	**0.004**	−0.37	−0.07
Chronic disease	Has only one/***Non*****e**	0.06	0.337	−0.07	0.20	−0.07	0.356	−0.21	0.08
	Has multiple/***Non*****e**	−0.34	0.070	−0.71	0.03	−0.37	**0.049**	−0.75	−0.001
	Has multiple/**Has only one**	−0.41	**0.030**	−0.77	−0.04	−0.31	0.110	−0.68	0.07
**II. Countries**									
Hungary	Slovakia/**Hungary**	−0.03	0.739	−0.21	0.15	−0.003	0.978	−0.19	0.18
	Czech Republic/**Hungary**	−0.13	0.143	−0.31	0.04	−0.14	0.139	−0.32	0.04
	Poland/**Hungary**	0.05	0.617	−0.14	0.23	0.11	0.286	−0.09	0.30
Slovakia	Hungary/**Slovakia**	0.03	0.739	−0.15	0.21	0.003	0.978	−0.18	0.19
	Czech Republic/**Slovakia**	−0.10	0.266	−0.28	0.08	−0.14	0.148	−0.32	0.05
	Poland/**Slovakia**	0.08	0.408	−0.11	0.26	0.11	0.256	−0.08	0.29
Czech Republic	Hungary/**Czech Republic**	0.13	0.143	−0.04	0.31	0.14	0.139	−0.04	0.32
	Slovakia/**Czech Republic**	0.10	0.266	−0.08	0.28	0.14	0.148	−0.05	0.32
	Poland/**Czech Republic**	0.18	**0.049**	0.001	0.36	0.24	0.011	0.06	0.43
Poland	Hungary/**Poland**	−0.05	0.617	−0.23	0.14	−0.11	0.286	−0.30	0.09
	Slovakia/**Poland**	−0.08	0.408	−0.26	0.11	−0.11	0.256	−0.29	0.08
	Czech Republic/**Poland**	−0.18	0.049	−0.36	−0.001	−0.24	**0.011**	−0.43	−0.06

*Coef, Coefficient; CI, Confidence interval; P, significance of statistical test; P < 0.05 significance; Reference groups are underlined. The multivariate analysis includes all demographic variables as confounding effects. The values in bold are significant*.

## Discussion

Since only the BMQ-Specific sub-questionnaire was used to map the opinion of those taking cholesterol-lowering drugs within the general population, it is not possible to compare the results with other BMQ studies. After comparing the answers of the participants from the Visegrad Group countries, a hierarchy could be observed considering the necessity of anticholesterol medical treatment in which Polish respondents got the highest score, followed by the Czech together with the Slovaks, while Hungarians got the lowest score. This means that, relatively, the Polish respondents believed the most that they need to take their cholesterol-lowering medication. On the other hand, the differences regarding concerns were less noteworthy, as the only significant value was found when comparing the answers of Polish and Czech participants.

All these suggest that while the participants from the Visegrad Group countries do not differ meaningfully regarding their fear of cholesterol-lowering treatments, the beliefs about benefits are the most pronounced among Polish and the least among Hungarian respondents. According to Rosenstock's widely used Health Belief Model (26), a person's belief about the costs of the behavior related to their health can be considered as the perceived risks of the behavior while the beliefs about the necessity can be considered as the perceived benefits. The perceived risk reduces while the perceived benefit increases the likelihood of the behavior, which is the adherence in this case (27). That means that the lower level of adherence among the Hungarian participants cannot be adequately explained along the perceived risks compared to other Visegrad Group countries. In addition, not only the perceived risk but also the perceived benefits are the lowest among the Hungarian respondents as well. This could explain why only 63.4% of statins were redeemed in 2012 in Hungary (28). The causal link between raised cholesterol and cardiovascular diseases is well-known. A possible factor behind the low perceived benefit and low perceived risk of cholesterol-lowering treatment of Hungarian participants may be the lack of knowledge of this link.

Finally, it is worth highlighting the limitations of this study. For example, despite striving to analyze representative samples, when narrowing down the original sample to only those who took cholesterol-lowering drugs, as seen in the descriptive statistics, the sample lost its representativeness. Thus, it is difficult to tell how much of these findings can be generalized. Furthermore, the questions regarding the financial status and health status were subjective and personal interpretations regarding these answers could have influenced the respondents. Also, despite asking a question about having a chronic disease, we did not specify what kind the respondents might have.

## Conclusion

Since no meaningful differences were observed regarding concerns for cholesterol-lowering drugs between the four Visegrad Group countries—with the exception of Czech responses in comparison to the Polish—the same approach could be used in all these countries to improve trust and thus improve medication adherence as well. To achieve this goal, we encourage establishing public health promotion programs designed for the improvement of health literacy and health behavior in all four countries. Also, successful policies and best practices should be shared between health experts to help each other in this endeavor. Finally, the underlying reasons regarding the differences identified in necessity should be explored in future studies.

## Data Availability Statement

The original contributions presented in the study are included in the article/supplementary material, further inquiries can be directed to the corresponding author/s.

## Ethics Statement

The studies involving human participants were reviewed and approved by the Scientific Research and Ethics Committee of the Medical Research Council (Hungary) and by the Ethics Committee of the Czech University Hospital Hradec Kralove (Czech Republic). The patients/participants provided their written informed consent to participate in this study.

## Author Contributions

KBí, VD, GB, and KBo conceptualized and designed the study. KBí acquisitioned funding, was important for intellectual content in terms of reviewing the manuscript, and supervised the study. VD and AN analyzed and interpreted the data. KBí, VD, ZF, GB, and KBo drafted the manuscript. All authors contributed to the article and approved the submitted version.

## Conflict of Interest

The authors declare that the research was conducted in the absence of any commercial or financial relationships that could be construed as a potential conflict of interest.
